# Rapidly Progressive Dementia Due to *Mycobacterium neoaurum* Meningoencephalitis

**DOI:** 10.3201/eid1005.030711

**Published:** 2004-05

**Authors:** George A. Heckman, Cynthia Hawkins, Andrew Morris, Lori L. Burrows, Catherine Bergeron

**Affiliations:** * Freeport Health Centre, Kitchener, Ontario, Canada; † McMaster University, Hamilton, Ontario, Canada; ‡ University of Toronto, Toronto, Ontario, Canada; § Toronto Western Hospital, Toronto, Ontario, Canada

**Keywords:** Rapidly progressive dementia, Mycobacterium neoaurum, Central nervous system, Meningitis, Arteritis, Protein 14-3-3

## Abstract

Dementia developed in a patient with widespread neurologic manifestations; she died within 5 months. Pathologic findings showed granulomatous inflammation with caseation necrosis, foreign body–type giant cells, and proliferative endarteritis with vascular occlusions. Broad-range polymerase chain reaction identified *Mycobacterium neoaurum* as the possible pathogen. Central nervous system infection by *M. neoaurum* may result in rapidly progressive dementia.

A few dementing illnesses are characterized by rapid cognitive decline and early emergence of neurologic signs. Causes include malignancy, vascular disorders, autoimmune disorders, and infections. We describe a patient in whom dementia associated with cerebellar, pyramidal, extrapyramidal, and bulbar manifestations developed; the patient died within 5 months. Postmortem examination showed chronic granulomatous meningitis and arteritis. Broadband polymerase chain reaction (PCR) identified the presence of DNA from *Mycobacterium neoaurum*.

## The Case

A 63-year-old woman was assessed for rapid functional decline over 2 months, with cognitive impairment, multiple falls, incontinence, and dependence for most basic daily activities. She could ambulate no further than a few meters despite assistance. Medical history included stroke 10 months previously with mild residual left-sided weakness, depression, rheumatoid arthritis, and hypertension. She smoked heavily, did not abuse alcohol or injection drugs, and had never received blood products. Medications included prednisone 5 mg daily, paroxetine, amlodipine, clopidogrel, estrogen, calcium, vitamin D, bromazepam, and acetaminophen. She was white and had spent all her life within the Great Lakes area of southern Ontario. She had once worked in a plant manufacturing leather components for automobiles, and her husband worked briefly as a meat packer. She had no family history of neurologic illness. She did not consume raw meat and had no contact with livestock but used sheep manure in her garden.

Cognitive testing showed impaired abstract thinking, memory, and attention but no affective or psychotic disturbance. She was afebrile with no nuchal rigidity. Speech production was reduced, aprosodic, and dysarthric. Cranial nerves were otherwise unremarkable. She exhibited hypomimia, limb rigidity with intermittent cogwheeling, and left arm dysmetria. No tremor or startle myoclonus was noted. Power was moderately reduced in all limbs. Reflexes were brisk with bilateral spontaneous ankle clonus of both ankles. Bilateral plantar responses were extensor. She could not walk unaided.

The patient was referred to a consultant and hospitalized. Complete blood count, blood urea nitrogen, creatinine, electrolytes, calcium, alkaline phosphatase, bilirubin, thyroid-stimulating hormone, and serum B12 were normal. Serum albumin was 28 g/L (normal 33–48 g/L), serum glutamic oxaloacetic transaminase 54 U/L (normal 5–40 U/L), serum glutamic pyruvate transaminase 58 U/L (normal 5–40 U/L), and erythrocyte sedimentation rate 74 mm/h. Serum antinuclear antibodies, extractible nuclear antibodies, antineutrophil cytoplasmic antibodies, Venereal Disease Research Laboratory test, and complement levels were unremarkable. Serologic tests for hepatitis B and C and enzyme-linked immunosorbent assay for HIV were negative. Electroencephalograph (EEG) demonstrated intermittent irregular slow delta waves in the right frontal and left temporal regions but no biphasic or triphasic waves. Magnetic resonance scan of the brain showed multiple areas of remote and recent infarction involving right frontal cortical and subcortical regions, pons and cerebellum, and right parasagittal frontal cortex. A diagnosis of recurrent strokes was made, but she continued to decline after discharge to a rehabilitation hospital. She became mute, immobile, and in need of complete assistance. She freely aspirated and was hypoxic. A lumbar puncture obtained shortly before her death showed cerebrospinal fluid (CSF) glucose of 4.8 mmol/L (normal 2.5–4.4 mmol/L), total protein of 0.33 g/L (0.15–0.60 g/L), and 8 x 10^6^ lymphocytes/L with no malignant cells. Bacterial, mycobacterial, and fungal stains and cultures and viral cultures were negative. Protein 14-3-3 (Centre for Research in Neurodegenerative Disease at the University of Toronto) was present in the CSF.

**Figure 1:**
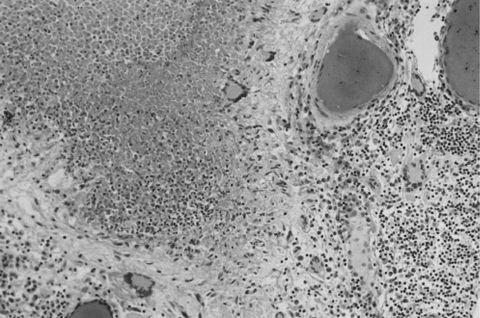
Meningeal infiltrate with caseation necrosis surrounded by giant cells. A nearby vessel is surrounded by a mononuclear cell infiltrate (occipital lobe, x250, stained with hematoxylin and eosin–Luxol-fast blue).

**Figure 2:**
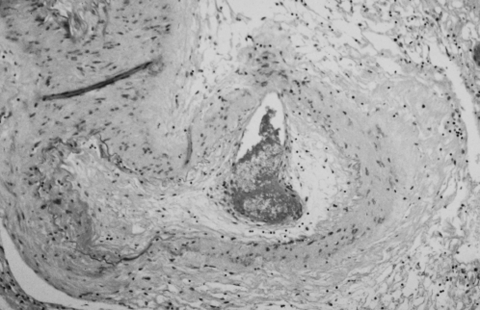
Proliferative endarteritis. The artery is stenotic and partially occluded by fibrous tissue. The residual lumen is almost completely occupied by recent thrombus (frontal lobe, x160, stained with hematoxylin and eosin–Luxol-fast blue).

The brain weighed 1,530 g. The circle of Willis was normal with no atheroma or occlusions. External examination showed focal areas of yellow exudate on the convexities and multiple bilateral infarcts affecting the cortices, pons, thalamus, middle temporal gyrus, and putamen. Histologically, some infarcts were bland while others were associated with a thick exudate characterized by granulomatous inflammation with caseation necrosis and foreign body–type giant cells (Figure 1). Numerous vascular occlusions with no atheroma were noted (Figure 2). A proliferative endarteritis was observed. In some areas, the process appeared resolved with extensive leptomeningeal fibrosis. Ziehl-Nielsen and auramine rhodamine stains failed to demonstrate mycobacteria. Gram stains, fungus stains, and prion protein immunostains (3H4, Prionics, Zurich, Switzerland) were negative. A moderate degree of diffuse arteriolosclerosis was observed. An occluded Charcot-Bouchard aneurysm was identified in the right temporal cortex.

Broad-range PCR identification was performed on frozen brain tissue by using the modified method of Heritz et al. (*1*). PCR (PerkinElmer, Foster City, CA) and lysis buffers were pretreated with 8-methoxypsoralen at a final concentration of 25 μg/mL and exposed to long-wave (360 nm) UV light for 15 min to destroy preexisting DNA contaminants. Tissue samples (approximately 2 mm^3^) were lysed in 50 μL 1x PCR buffer containing 1% polyoxyethylene 10 lauryl ether and 200 μg/mL proteinase K at 56°C overnight. Lysate, in 1- and 5-μL aliquots, was used as template for PCR amplification with universal primers for bacterial 16S rDNA (515FPL and 13B [*2*]). PCR mixture without lysate was used as a negative control, and a cloned 16S rDNA amplification product was used as a positive control (20 ng DNA). The PCR protocol involved a 3-min hot start at 95°C, followed by addition of primers at 50 pmol per 50-μL mixture. Thirty cycles of PCR were performed (94°C, 45s; 55°C, 45s; 72°C, 1 min) followed by a final extension step at 72°C for 10 min, and the amplification products were stored at 4°C. The PCR products were separated on a 1.5% agarose gel and visualized on a UV transilluminator after being stained with ethidium bromide (0.5 g/mL) for 15 min at room temperature. Amplification products were cloned into the plasmid vector pCR2.1TOPO (Invitrogen, Burlington, Ontario). Cloned products were fingerprinted by endonuclease digestion with *Taq*I or *Rsa*I, and unique clones were sequenced by using dye terminator methods (ABI PRISM BigDye Terminator Cycle Sequencing Kit, Applied Biosystems, Foster City, CA). The resulting DNA sequences were identified by comparison with the nonredundant database at the National Center for Biotechnology Information (*3*) and showed a match of 577 of 581 nt (99%) to *M. neoaurum* (GenBank accession no. AF268445).

## Conclusions

*M. neoaurum*, first described in 1972, belongs to the *M. parafortuitum* complex of the genus *Mycobacterium* (*4*). *M. neoaurum* is a rapidly growing scotochromogen that is found in soil. Eight cases of human infection from *M. neoaurum* have been described in the English-language literature (*5*–*11*). Six of these cases were associated with central venous catheters, one was associated with intravenous drug use, and another involved urinary isolation of *M. neoaurum* during an investigation of a catheter-associated urinary tract infection. None of these cases was fatal. CNS infection has not been previously described.

Several features of this case suggest that infection caused by *M. neoaurum* was responsible. First, the patient declined rapidly, which is unusual in common dementias. Second, a mild CSF lymphocytic pleocytosis is consistent with an infectious or inflammatory CNS disorder, though rare occurrences have been reported in pathologically confirmed Creutzfeldt-Jacob Disease (CJD) (*12*). Third, histologic examination demonstrated a caseating granulomatous process with accompanying endarteritis, consistent with mycobacterial infection. *M. neoaurum* is relatively difficult to culture; therefore, inability to cultivate the organism or visualize it on Ziehl-Nielsen and auramine rhodamine stains is not unexpected.

The patient had no known risk for parenteral exposure to bloodborne pathogens and was not apparently immunocompromised. Although the epidemiology of her case suggests mycobacterial infection through direct exposure, the mode of acquisition is speculative.

The patient was diagnosed with CJD. In patients with rapidly progressive dementia, probable CJD may be diagnosed if myoclonus or typical EEG tracings are present, according to Brown et al. (*13*). Possible CJD is diagnosed when rapidly progressive dementia is associated with a movement disorder or periodic EEG activity. Masters proposed that probable CJD may be diagnosed in patients with rapidly progressive dementia, biphasic or triphasic waves on EEG, and at least two of myoclonus, visual or cerebellar symptoms, pyramidal or extrapyramidal signs, and akinetic mutism (*14*). Possible CJD is diagnosed when typical EEG findings are absent. Recent efforts to improve diagnostic accuracy for CJD have focused on measuring CSF protein 14-3-3. Thought to reflect neuronal injury, reported sensitivities and specificities of CSF protein 14-3-3 for CJD in patients with rapidly progressive dementia are 84%–96% and 87%–100%, respectively (*15*–*21*). The Masters’ criteria have been revised to reclassify possible CJD as probable if CSF protein 14-3-3 is present (*18*). In the case described, revised Masters’ criteria for probable CJD and Brown criteria for possible CJD were met. The false-positive rate of CSF protein 14-3-3 for CJD can be as high as 12%, however, with other causes including vascular disorders, infectious encephalitis, anoxia, malignancy, and even Alzheimer’s, Lewy body, and frontotemporal dementias (*15*,*17*–*21*). In this patient, the presence of CSF protein 14-3-3 likely reflected ischemic injury mediated by the proliferative arteritis.

In summary, we have described a case of rapidly progressive dementia with prominent neurologic features attributable to chronic granulomatous meningitis and arteritis. Despite negative stains and cultures, the identification of DNA from *M. neoaurum* suggests that this case may represent the first reported CNS infection from this organism, as well as the first documented fatality. The absence of any clear mode of infection or predisposing risk factors for developing such a devastating infection is unusual. This case highlights the difficulties in achieving a causative diagnosis in patients with rapidly progressive dementia. CSF protein 14-3-3 does not entirely rule out potentially treatable causes, and in this case an angiogram would likely not have been able to differentiate inflammatory from infectious vasculitis. More definitive diagnostic methods are required, including a possible role for PCR analysis of CSF samples, as well as earlier consideration of biopsy.
